# Supplementary material for: Are carbon emissions trading and green financial instruments synergistic? -Comprehensive quantitative research based on content analysis
Source: PLoS One. 2024 Mar 7;19(3):e0298601. doi: 10.1371/journal.pone.0298601 (PMC10919601; doi:10.1371/journal.pone.0298601)
Supplement: S1 Data — (ZIP) [file pone.0298601.s001.zip › Data/地级市Z_F工作报告.zip!/─■╧─/╬Γ╓╥╩╨/└┤╫╘╣1⁄2╓┌║┼í╛┬φ┐╦╩2╛▌═°í┐_2019.docx]

各位代表：

现在，我代表市人民政府，向大会报告政府工作，请予审议，并请市政协委员和其他列席人员提出意见。

2018年工作回顾

2018年，是全面贯彻落实党的十九大精神的开局之年。一年来，我们以习近平新时代中国特色社会主义思想为指导，深入学习贯彻十九大和习近平总书记视察宁夏重要讲话精神，全面落实自治区第十二次党代会部署要求，在自治区党委、政府和市委的坚强领导下，在市人大、政协的监督支持下，坚持稳中求进工作总基调，认真贯彻高质量发展要求，坚持不懈抓重点、补短板、强弱项，坚定不移推进稳增长、促改革、调结构、惠民生、防风险各项工作。全市各族人民在习近平总书记“建设美丽新宁夏、共圆伟大中国梦”题词的感召下，在改革开放40周年、自治区成立60周年辉煌成就的鼓舞下，热爱祖国、建设家乡的热情更加高涨。我们以高度的政治自觉，扎实推进中央巡视、环保督察“回头看”和国务院大督查反馈意见的整改落实，发展质量稳步提升，生态环境明显改善，发展基础更加牢固，朝着实现经济繁荣、民族团结、环境优美、人民富裕，确保与全国全区同步建成全面小康社会目标又迈出了坚实有力的一步。全年实现地区生产总值534.5亿元，增长6.6%；一般公共预算收入34.6亿元，同口径增长8%；社会消费品零售总额119.2亿元，增长5.7%；城镇居民人均可支配收入27478.1元，增长8.3%，农村居民人均可支配收入12045.4元，增长10.4%；居民消费价格同比上涨2.5%。

坚持稳中求进，经济运行平稳向好。面对下行压力，精准靶向施策，及时出台普惠金融提升、沿黄生态经济带建设等7个行动计划，推进落实创新驱动、工业对标提升、加快会展业发展等19个针对性较强的措施方案，有力推动了全市经济持续健康发展。实施5000万元以上工业项目93个，伊利乳业液态奶生产线、中国自动化绿色精密铸锻产业园等项目加快建设，华能大坝四期扩建等项目建成投产。积极推进园区整合优化，9个工业园区整合优化为5个，理顺了体制，整合了资源，激发了活力。大力支持企业降本增效，实施高沙窝集中区8公里供水专线、金积工业园区集中供热供汽等17个低成本化改造项目。认真落实自治区“降成本30条”等政策措施，争取各类惠企资金2.56亿元。严格落实市级领导包抓责任制，盘活处置僵尸企业15家，整治散乱污企业89家，淘汰落后产能117.6万吨。加快传统行业绿色提标改造，伊利乳业创建为国家级绿色工厂。全市单位工业增加值能耗同比下降4%。新增规上企业34户、产值过亿元企业10户，规上工业增加值增长7.5%。全市粮食播种面积307.5万亩，实现农业增加值70亿元，增长4.2%。奶牛存栏24.1万头，肉牛、肉羊、黑毛驴饲养量分别达到48万头、660万只和3万头。认证“三品一标”农产品28个，新增市级以上农业龙头企业40家、农民专业合作社23个、家庭农场28个，新建富硒农产品检测中心、富硒功能农业博士工作站各1个。新增酿酒葡萄、文冠果等经济林13.8万亩，总面积94.72万亩，新建苹果、枸杞、酿酒葡萄标准化示范基地6个。盐池滩羊入选首批中国特色农产品优势区，同心银柴胡种植基地入选全国特色中药材种植基地，吴忠亚麻籽油成为全国首个亚麻籽油地理标志产品。塞外香、君星坊分别入选全国大米加工50强企业和全国亚麻籽油加工10强企业榜单。培育发展休闲农业经营主体130个。组建红寺堡壹加壹肉牛等产业化联合体16家。我市被评为全国平安农机示范市，利通区、青铜峡市创建为主要农作物生产全程机械化示范县，盐池县被确定为第三批国家高效节水灌溉示范县，吴忠国家农业科技园区被认定为国家有机食品生产基地建设示范区。万达广场等商贸项目投入运营，宁夏德昌铁路物流中心等项目进展顺利。新建乡村电商服务站16家，总数达到451家，覆盖率90.2%。光耀美食街等特色街区建成运营，“吴忠早茶”“吃在吴忠”的美誉度和影响力进一步提升。大力提升35个优质特色产品展示展销中心运营水平，全年实现销售额3.12亿元。实现进出口总额8亿元，同比增长20%。全域旅游示范市创建扎实推进，接待游客和旅游收入同比增长12%和15%。争取项目1005个，批复资金148.9亿元，实施招商引资项目166个，到位资金309.7亿元。全年经济运行稳、就业形势稳、物价涨幅稳、社会大局稳，内生动力显著增强，质量效益继续向好。

坚持底线思维，风险防控措施有力。强化政府债务管理，建立“借、用、还”统一的债务管理机制，未发生违法违规举债融资行为。加大市本级政府债务化解力度，历史形成的政府系统内债务全部实现债券化，由风险预警地区降为提示地区。出台《吴忠市防范化解金融风险实施方案》，制定24条有效措施，确保不发生区域性、系统性风险。组建债委会11家，逾期90天以上贷款纳入不良管理。完善金融监管机制，严厉打击非法集资、金融诈骗等各类违法犯罪行为。全市各项贷款余额616.6亿元，同比增长5.2%，新增贷款30.5亿元。依法打击违规销售、违规交房等行为，从源头防控房地产市场风险。深入落实促进房地产市场平稳健康发展的10条政策措施，房地产销售面积140万平方米，库存下降10%。建立完善市、县、乡三级食品药品安全监管机制，妥善应对处置问题疫苗等风险隐患。市食品检测中心加快建设，建成食品及食用农产品快检室64个，“明厨亮灶”覆盖率达98%。

坚持精准施策，脱贫攻坚成效显著。实施打赢脱贫攻坚战三年行动，脱贫销号25个村，减少贫困人口34693人，贫困发生率由6.54%下降为2.74%。加快盐同红交界处集中连片贫困地区发展，投入48亿元，实施公共服务均等化、基础设施建设、生态修复保护等270个项目。盐池县成为全区首个脱贫摘帽县，综合贫困发生率降到0.66%、群众满意度97.71%，获2018年全国脱贫攻坚组织创新奖。红寺堡文化扶贫“宁夏样本”代表西部地区在全国做了经验交流。同心县扶贫车间被评为2018民生示范工程，预旺镇贺家塬村第一书记丁海燕荣获全国脱贫攻坚贡献奖。争取闽宁协作项目资金1亿元，落实央企帮扶资金4620万元，实施精准到村到户项目61个。培育发展扶贫龙头企业29家、专业合作社71家、巩固提升产业扶贫示范村22个，建设扶贫车间34家。续建移民住房1000套，搬迁安置移民1108户4589人，“十三五”异地扶贫搬迁任务全面完成。累计投放扶贫小额贷款25亿元，贷款覆盖率90%，户均4.9万元。全面落实教育资助政策，开展“控辍保学”专项整治，249名孩子重回校园。健康扶贫“一站式”结算覆盖所有贫困人口，大病医疗救助惠及8864名困难群众。中央第八巡视组反馈脱贫攻坚领域的7个方面24个问题完成整改20个，需要长期整改的4个。

坚持铁腕治污，生态环境稳定改善。加快创建国家森林城市，完成营造林35.6万亩，森林覆盖率达到16.5%。耕地保有量459.55万亩，永久基本农田保护面积368.22万亩，获全区耕地保护一等奖，利通区、盐池县、同心县土地节约集约利用被国务院通报表扬。深入开展“蓝天碧水净土” 和“绿盾2018”自然保护区清理整治专项行动，中央第八环保督察组反馈问题得到切实整改，中央第二环保督察组“回头看”转办的34批250件294个问题全部办结。罗山自然保护区内38台风电机组全部拆除，哈巴湖自然保护区人类活动点位彻底清理。加大环保执法力度，立案查处186件，处罚金额1675万元。市环保局被授予全国环境保护系统先进集体称号。大力实施燃煤锅炉“清零”行动，利通区、青铜峡市城市建成区内燃煤锅炉全部淘汰停用，其他县（区）城市建成区内20蒸吨/小时以下燃煤锅炉全部淘汰。划定全区首个城市禁煤区，率先在全区推行餐饮企业和商户“煤改气”，完成改造1128家。全年空气质量优良天数262天，PM10、PM2.5平均浓度同比分别下降9.9%和15%。全面落实河（湖）长制，全市9个生活污水处理厂全部达到一级A排放标准，城市黑臭水体全面消除，金积饮用水水源地及3个地下水监控点位水质保持稳定，清水沟、南干沟入黄水质稳定提升为Ⅳ类，黄河吴忠出境断面水质一直保持在II类优水质。治理水土流失312.9平方公里、盐碱地12万亩。

坚持改革创新，发展活力不断增强。深化“多证合一”，率先在全区推行个体工商户简易登记、豁免登记改革，开展“口头申报、当场领照”免填单登记新模式。企业登记实行“审核合一、一窗通办”，办事效率提高35%以上。建立市场监管、公安、税务等跨部门协作机制，企业开办时间由11个工作日压缩到5个工作日。全市新增市场主体6887户，增长7.1%，总量达到103308户，位居全区第二。推行“互联网+政务服务”，推进“不见面、马上办”审批服务，81.7%的政务服务事项实现可不见面办理，39个事项可不出村（社区）办理，公共资源交易实现“最多跑一次”。全区首个市场化运作的农村产权交易机构吴忠农村产权交易中心开业运营，挂牌土地13978亩，交易金额7125万元。同心县累计发放农村承包土地经营权抵押贷款10.4亿元，居全区五个试点县首位。稳步推进国企混合所有制改革，城投公司参股宁夏德阅基金管理，办理助贷业务37亿元。宁夏水投吴忠水务公司挂牌成立。全市国有企业资产总额171亿元，实现营业收入10亿元，增长25%。完善医院法人治理体系，落实公立医院取消药品加成亏损分担机制，深化按病种收付费改革，动态调整市县级公立医院诊疗服务价格。率先在全区出台优化营商环境25条措施，大力支持民营企业发展，民营经济增加值占全市GDP的51.5%。认真落实创新驱动行动计划，R&D投入4.45亿元，增长45.4%，增速居全区五市第一，投入强度达到0.88%。专利申请量952件，每万人有效发明专利拥有量达到2.13件。新增国家高新技术企业8家、科技型中小企业41家、星创天地5家，自治区科技型中小企业34家、农业高新技术企业5家、科技小巨人企业9家。法福来食品等4家企业被评为自治区级企业“双创”平台，获批建设全区首个中国智能装备制造（仪器仪表）产业知识产权运营中心，盐池县被科技部确定为全区唯一一家“首批创新型县”，金积工业园区成功创建为国家级创业创新示范基地，吴忠中小企业公共综合服务窗口平台创建为国家级中小企业公共服务示范平台。

坚持统筹推进，区域城乡协调发展。制定《推进银川都市圈建设行动计划（2018—2020年）》《加快推进利青同城化发展实施方案》，开工建设滨河大道与黄河大桥立交匝道等33个融入银川都市圈重点项目，与银川二十一小等学校建立教育合作共同体9个。青铜峡至银川正源街快速通道、国道344线吴忠至灵武段公路基本完工。全年实施城乡建设项目150个，完成投资109亿元，吴忠奥林匹克体育中心、城市数据运营中心等建成运行。实施市区“城市双修”工程，改造道路19条25.7公里、人行道10万平米、楼体140余栋，新建城市绿道20公里。建成市区东部地下综合管廊9.32公里。综合改造老旧安置小区7个，新建棚户区改造房7527套。购置纯电动公交车30辆，优化城市公交线路3条。建设美丽小城镇7个、美丽村庄示范点40个，改造农村危房7484户、农厕8661个，新建农村公路542公里。利通区东塔寺乡石佛寺村入选全国传统村落名单，市住建局被评为全国住房城乡建设系统先进集体。认真开展“大棚房”清理整治，拆除违法违规建筑设施586个（栋）。颁布《吴忠市城乡容貌和环境卫生治理条例》，利通区、红寺堡区、青铜峡市、盐池县推行农村环境治理市场化运营模式，青铜峡市荣登“2018年中国最美县域”榜单，农村人居环境持续改善。

坚持民生为要，社会事业全面进步。高质量办结10项30件民生实事。新增城镇就业1.47万人，城镇“零就业”家庭动态消零，城镇登记失业率3.21%，农村劳动力转移就业20.8万人。发放农村妇女创业担保贷款2.9亿元，为4756名妇女创业提供了资金支持。城乡居民基础养老金标准由170元提高到193元，80岁以上老年人实现“零缴费”参保。市县财政安排资金4214万元，广泛开展扶危济困送温暖活动。康乐养老服务中心被确定为国家第二批公办养老机构改革试点单位。利通区古城村被评为全国首批农村幸福社区建设示范单位。我市再次荣获慈善城市奖，王兰花被授予第十届“中华慈善奖”慈善楷模。红十字会“三救三献”工作扎实推进，救援救护能力有效提升。投资1.29亿元，建设残疾人康复中心6所、托养中心4所。培育自治区青年拔尖人才19人，柔性引进高层次人才93人，累计享受国务院特殊津贴9人。制定落实吴忠市《深化教育体制机制改革实施意见》《全面深化新时代教师队伍建设改革行动计划》，教育教学质量显著提高。投资3.5亿元，新建改扩建学校62所，基本消除“超大班额”。实施“互联网+教育”和集团化办学，组建教育共同体71个。高考二本以上上线率提高3.6个百分点。青少年科技创新再创佳绩，172件作品获得第33届全国青少年科技创新大赛奖项。大力推进“互联网+医疗健康”，深化与首都医科大学、北京302医院等医疗机构的合作，组建家庭医生签约团队291个。市人民医院、盐同红三县区公立医院与贫困地区13所乡镇卫生院建立医疗联合体。医保按病种付费数由28个扩展到125个。全面落实疾病防控措施，及时有效处置了禽流感、乙脑、皮肤炭疽等疫情。利通区、青铜峡市通过国家慢性病综合防控示范区复审，盐池县创建为全国妇幼健康优质服务示范县。吴忠美术馆、红寺堡文化馆建成运行，吴忠图书馆、同心图书馆被评为国家一级馆。启动新一轮全国文明城市创建工作。成功创建为国家公共文化服务体系示范区。组织“文化惠民送戏下乡”演出605场次、“滨河百姓大舞台”广场演出71场次。成功举办第七届宁夏黄河金岸国际马拉松赛、第五届全国山地旅游自行车赛等赛事，2018年全国沙滩排球巡回赛（宁夏吴忠站）荣获最佳赛区奖、贡献奖。鸽子山遗址考古研究项目获中国考古学会田野考古奖一等奖。李进祯荣登“中国好人榜”，李俊义等3个家庭被评为2018年全国“最美家庭”，马靖廷等3个家庭被评为第十一届全国“五好家庭”，微电影《李进祯》获第六届亚洲微电影艺术节一等奖。大力提升气象综合监测服务能力，升级改造骨干网站15个，优化布局特色农业等实景观测站点30个。加快推进档案信息化建设，管理服务质量进一步提高。全年归集住房公积金10.5亿元，同比增长7.6%。

坚持依法治市，社会大局和谐稳定。深化民族团结进步“五项特色教育”，常态化开展“回汉一家亲”“社区邻里节”等创建巩固提升活动。坚持宗教中国化方向，积极稳妥推进宗教领域突出问题整治，推行宗教场所财务民主管理第三方代理记账模式。严格落实安全生产责任制，建立健全安全生产“一票否决”、党政领导干部责任制等长效机制，立案查处77家违法违规企业，生产安全事故起数和死亡人数同比下降31%、30%。深入推进“七五”普法，深化人民调解“四张网”建设，市、县、乡三级信访事项人民调解组织实现全覆盖。认真做好退役军人服务管理工作，建立“四级”包联机制，妥善安置577人，保障维护退役军人合法权益。办理中央第八巡视组移交我市群众信访件478件、办结率98%。全力抓好市委巡察反馈问题整改，215个问题已完成整改205个，整改率94.9%。扎实推进平安吴忠建设，深入开展扫黑除恶专项斗争，打掉涉恶团伙12个，全市治安案件、八类主要刑事案件、抢夺案件、毒品案件同比分别下降5%、2.9%、34.8%和45.3%。自觉接受人大法律监督、政协民主监督，支持人大建设代表之家、政协实施民主协商计划，16个部门主动接受人大工作评议，40个部门聘请了特约监督员。办理人大代表议案建议64件、政协委员提案198件。主动接受新闻舆论和社会公众监督，广泛听取民主党派、工商联、无党派人士意见建议。加快建设失信联合惩戒机制，积极协助破解执行难题，法院案件执结率同比提高9.02%。在全区率先出台《关于支持检察机关开展公益诉讼工作的意见》，公益诉讼案件240件，有效维护了公共利益。支持工会按规定保障干部职工享有福利待遇，新建劳模创新工作室13家，帮扶救助困难职工9666人次，市总工会被评为全国“安康杯”竞赛优秀组织单位。常态化开展“关爱行动”等志愿服务活动，全市实名注册志愿者17.2万人。整合开通12345便民服务热线，我市位列全国12345热线服务质量监测排名榜第十七名、宁夏第一名。市交通运输局被评为全国交通运输行政执法评议考核优秀执法单位。积极开展预算项目绩效评价，财政预算公开率达到100%。加强审计监督，完成审计项目40个，查出问题金额4.8亿元，移送处理5项。加大政务督查力度，及时跟踪督办民生实事等重点工作200余项，有效推进了各项决策部署落实到位。坚持把党的领导贯穿政府工作始终，进一步增强“四个意识”，坚定“四个自信”，坚决做到“两个维护”。严格落实中央八项规定精神，坚持不懈整治“四风”，廉洁自律意识不断增强，工作作风持续好转，政府效能明显提升。

各位代表，回顾刚刚过去的不平凡的一年，我们感恩党中央、国务院和自治区的亲切关怀，感谢市人大、政协的监督支持，感激全市143万各族人民群众的努力拼搏。在此，我代表市人民政府，向奋战在各条战线的广大干部群众，向人大代表、政协委员、各民主党派、工商联，向离退休老同志和社会各界人士，向驻吴人民解放军、武警部队、公安民警和消防救援队伍，向中央及区属驻吴单位，向关心支持吴忠发展的国家部委、自治区厅局、友好城市、政府顾问和外商、港商、台商表示崇高的敬意和衷心的感谢！

认真对标对表，我们工作上还存在许多不足和差距，发展中也有不少困难和问题，主要是：发展质量不高，产业层次较低，工业转型升级困难，现代服务业发展缓慢，主要经济指标低于预期；大项目不多、好项目较少，经济增长基础不牢固，新的增长点不够多；山川、城乡、区域发展不均衡、不协调问题突出，攻坚深度贫困任务艰巨；科技创新能力不强，科技投入仍然不足，各类创新人才短缺；教育、医疗、文化、就业等公共服务水平与人民群众日益增长的美好生活需要还有较大差距；政府职能转变还需继续加快，管理服务水平有待进一步提高，等等。这些矛盾问题的交织迭加，困扰制约着吴忠高质量发展。我们将坚持问题导向，着力破解难题，以更加坚决的态度、更加有效的措施、更加务实的作风，努力把各项工作做实做细做好，不辜负全市人民的期望和重托。

2019年主要工作

2019年是中华人民共和国成立70周年，是全面建成小康社会关键之年，做好今年工作意义重大。当前，我国发展仍处于并将长期处于重要战略机遇期，经济长期向好态势没有改变。习近平总书记指出的我国面临加快经济结构优化升级、提升科技创新能力、深化改革开放、加快绿色发展、参与全球经济治理体系变革带来的“五个新机遇”，为我们抢抓机遇、用好机遇提供了行动指南和重要遵循。新时代改革开放再出发、“一带一路”建设深入推进、新一轮西部大开发深入实施，为我们注入了强大发展动力。习近平总书记“建设美丽新宁夏、共圆伟大中国梦”的重要题词，为新时代宁夏发展擘画了美好蓝图，赋予了新的历史使命。总的看，形势总体有利，机遇就在眼前。只要我们始终坚持党的领导，牢牢把握高质量发展要求，认真贯彻供给侧结构性改革“巩固、增强、提升、畅通”八字方针，坚决打好“三大攻坚战”，全面落实“三大战略”和石泰峰书记对吴忠提出的“四新”要求，实字当头，干字为先，就一定能应对各种困难挑战，高质量发展之路就一定会越走越宽，全市经济社会发展一定会再上新台阶。

今年政府工作的总体要求是：以习近平新时代中国特色社会主义思想为指导，全面贯彻党的十九大和十九届二中、三中全会及中央经济工作会议精神，坚持稳中求进工作总基调，坚持新发展理念，坚持推动高质量发展，坚持以供给侧结构性改革为主线，坚持深化市场化改革，扩大高水平开放，深入落实自治区第十二次党代会、自治区党委十二届六次全会和市委五届七次全会部署，按照石泰峰书记对吴忠提出的“四新”要求，继续打好“三大攻坚战”，认真落实“三大战略”，统筹推进稳增长、促改革、调结构、惠民生、防风险工作，干在实处、走在前列，为实现经济繁荣民族团结环境优美人民富裕、与全国全区同步建成全面小康社会打下决定性基础，为“建设美丽新宁夏、共圆伟大中国梦”作出新的更大贡献，以优异成绩庆祝中华人民共和国成立70周年。

经济社会发展主要预期目标是：地区生产总值增长7%左右，一般公共预算收入同口径增长7%，城镇调查失业率控制在5.2%左右（登记失业率控制在4%以内），居民消费价格涨幅控制在3%左右，贫困人口减少2.3万人，城乡居民人均可支配收入分别增长8%、9%，万元生产总值能耗和四项主要污染物指标完成自治区下达任务。

吴忠是欠发达地区，只有在确保高质量发展的同时努力提升发展速度，才能缩小与先进地区的差距。确定这样的目标，是市委、政府综合考虑宏观经济环境、自身基础条件和全面建成小康社会进程，权衡各方面因素确定的，既遵循了我市经济中长期发展规律，又反映了现阶段增长优势潜力。完成上述预期目标，保持经济稳中有进、进中向好发展，需要充分挖掘发展新动能，加快培育新的增长点。重点抓好以下工作。

一、更加注重转型升级，着力推动高质量发展

始终保持高质量发展的坚定信心和战略定力，开展高质量发展课题研究，深入推进供给侧结构性改革，进一步推进转型升级，努力实现更高质量、更有效率、更加公平、更可持续的发展。

加快工业提质扩量。全面落实工业对标提升转型发展措施，加快建设春升源二期年产2万吨调味品、宁疆炭材料年产10万吨煤基活性炭等80个重点工业项目，力争规模以上工业增加值增长7.5%，新增规上企业20户、“专精特新”中小企业20户。加快改造提升传统产业，实施金世纪包装环保节能绿色生产等30个技术改造项目。稳步推进企业优胜劣汰，加快“僵尸企业”出清，探索企业退出有效机制，化解淘汰过剩和落后产能136万吨。大力发展先进制造业，加快建设中国自动化绿色精密铸锻产业园、黄河电焊机自动化机器人焊接与智能化控制设备应用等项目，全力打造装备制造产业聚集区。实施现代纺织业提升行动计划，建设兴民纺织二期、帕罗羊绒纱深加工等项目，力争恒丰瑞斯特纺纱等项目投产达效。提升能源化工产业发展水平，重点实施新珂源天然气液化等项目，不断提高产品技术含量和附加值。深入推进园区整合优化，培育壮大主导产业，实施太阳山开发区动力岛等14个低成本化项目，建设智慧园区、科技孵化器等公共服务平台，完善气、水、电、路等基础设施配套，制定细化园区土地集约利用、投资产出强度标准，引导园区提高集聚力和承载力。

加快农业提档增效。加快构建现代农业“三个体系”，农业增加值增长4.5%。统筹调整种植业生产结构，优质粮食面积稳定在100万亩左右，新增酿酒葡萄、有机枸杞、文冠果等经济林8万亩，种植黄花菜16万亩。大力支持利通区创建国家现代农业产业园，做实做响同心县“肉牛养殖大县”品牌，实施红寺堡壹加壹肉牛产业链等项目。新增奶牛2万头、肉牛3万头、肉羊10万只、黑毛驴1万头。累计建设富硒农产品生产基地70个、培育富硒品牌25个，富硒作物面积达到23万亩。努力开拓国内高端消费市场，打造高品质食材供应基地。新增滩羊、肉牛、蔬菜、枸杞、黄花菜产业化联合体5个、自治区级农业产业化龙头企业5家。加强农业品牌建设，打响“青铜峡大米”“吴忠瓜菜”等地理标志品牌，新培育“三品一标”农产品15个。实施农业新型经营主体培育行动，新增农民专业合作社和家庭农场50个。打造休闲农业和乡村旅游精品工程，实施盐池县一二三产融合先导区创建等项目。建立完善农产品质量追溯、农作物病虫害预测预报体系，加大疫情防治力度。加强防灾减灾、气象服务等工作，扩大农业保险覆盖面，增强农业抗风险能力。

加快服务业提速升级。大力开展“服务业提升年”活动，推动商贸、旅游等产业融合转型，力争第三产业增加值增长8%。加快创建服务业集聚区，打造健康养生、现代金融、文化创意等10个服务业集聚区。加快泰富能源物流等项目建设，打造智慧物流公共服务信息平台，拓展现代物流服务能力。持续扩大“吃在吴忠”品牌优势，深入推进“两区、三街、四广场”餐饮品牌提升、名店名厨进驻等工作，不断增强集聚辐射带动能力。实施“十大促消行动”，全年举办各类促销活动30场次以上。举办全国航空航天模型锦标赛、全国跳伞锦标赛等一批知名体育赛事活动，培育和发展文化产业，进一步提升吴忠知名度。组织实施金积农贸市场、盐池综合市场、河西农贸市场等升级改造项目和菜篮子工程等民生项目，加快建设市、县、乡三级邮政快递服务体系。大力发展电子商务，确保红寺堡区、青铜峡市示范县项目通过国家验收，电商交易额达到15亿元以上。积极推进全域旅游示范市创建，力争青铜峡黄河大峡谷创建成5A级旅游景区，加快盐州古城历史文化旅游区5A级景区创建步伐。全年接待游客和旅游收入分别增长15%和20%以上。

防范化解重大风险。严格控制、积极化解政府债务，完善追收、消化、管理措施，坚决杜绝违规举债。落实稳金融政策，加强金融组织监管和金融类活动风险排查，有效防控和化解银行不良资产。创优金融生态环境，实施“金融+”工程，推进普惠金融行动，扩大单户授信1000万元以下小微企业贷款。加快建设金融科技服务创业园，全年引进金融机构2家以上，挂牌上市企业5家以上。发挥制造业、扶贫产业基金作用，力促服务业、奶产业等9支政府担保基金撬动不低于5倍的银行贷款。制定出台商品房预售资金监管和二手房交易资金监管办法，合理制定房地产开发用地供应计划，重点实施碧桂园等40个292万平方米房地产项目，保障群众住房改善性需求。规范商品房销售、交付行为，加大违法违规行为查处力度，促进房地产市场规范有序、健康发展。

二、更加注重项目建设，稳定扩大有效投资

坚持把稳投资作为重要抓手，全力引、积极争、加快建，为经济社会发展提供有力支撑。

认真谋划储备项目。紧盯中央、自治区政策导向和投资方向，抢抓国家“稳投资、保持基础设施领域补短板力度”机遇，积极谋划助推脱贫攻坚、产业发展等方面的大项目好项目，努力形成接连不断、滚动实施的项目储备机制，力争全年项目储备规模达到600亿元以上。加大上争资金力度，全面梳理财政资金、项目申报目录，建立争取资金项目台账。围绕融入银川都市圈建设等重点领域，积极争取资金，补齐投资缺口，夯实投资基础，全年上争资金150亿元以上。扩大民间资本投资范围，积极引导民营企业参与PPP项目开发建设。

加快推进项目建设。做实做细银川都市圈城乡东线供水等396个计划新开工项目的前期准备工作，力促春季项目开复工率达到60%以上。重点实施瑞科能源30万吨丙烷脱氢等285个产业项目、清水河同心段综合治理等98个农林水项目、太阳山至红寺堡公路等43个交通项目、红寺堡区第五中学等52个社会事业项目、盐池县城市道路及排水等82个城镇基础设施项目，全年完成投资430亿元以上。完善重点项目市级领导包抓和稽查督导机制，积极协调解决项目建设中的困难问题，确保伊利1700吨液态奶等164个项目竣工投产。

扩大招商引资成果。紧盯京津冀、长三角、珠三角等产业转移重点地区，围绕装备制造、现代农业、生态纺织、新材料新能源和生物医药等产业不断补链、强链、延链，以新企业催生新动能。落实自治区“高质高效招商引资年”活动要求，创新招商引资方式，探索委托招商、中介招商、代理招商等市场化运作方式，提升招商专业化水平，力争引进10亿元以上项目10个，20亿元以上项目5个，到位资金增长8%以上。

三、更加注重统筹协调，积极融入银川都市圈

认真落实《吴忠市空间规划》，加快利青一体化进程，在融入银川都市圈上取得新突破。实施城乡建设项目159个，促进城乡融合发展，推进以人为核心的新型城镇化。

主动融入银川都市圈。协调银西高铁、城际铁路、京藏高速、银百高速、乌玛高速、银昆高速6个重大项目有序推进，实施滨河大道改造等工程，确保青铜峡至银川正源街快速通道和国道344线吴忠至灵武段建成通车，构建融入银川都市圈大交通体系。加快打造都市圈先进装备制造业联盟，积极构建贺兰山东麓葡萄酒产业链。深化与自治区人民医院、宁夏医科大学总医院的战略合作，推进专科诊疗中心、特色专科联盟、专业技术实训基地建设，扩大卫生合作辐射效应，着力提升医疗服务水平。积极开展以教研共同体为载体的教学研讨、学术课题交流，支持6所职业院校与自治区高等院校建立职教联盟，承接共享银川市优质教育资源。加强区域环境保护，完善联防联治机制，推进都市圈内污染防治一体化，打造都市圈绿色生态廊道。

加快城市提质扩容。编制《国土空间规划》，强化规划“一张网”管理。开通吴忠高铁站至青铜峡快速城际公交线路，规划建设利青黄河大桥、黄河利青段旅游航线。稳步推进出租车行业改革和网约车政策落实，建立完善交通信息化管理平台。完善利青同城双向转诊绿色通道，巩固利青同城15分钟卫生应急救援保障圈。加快高铁吴忠枢纽站建设，打造高铁片区经济集聚区。新建市区棚户区改造500套、续建6708套。实施胜利西路、朝阳东街等10条10.5公里市政道路建设工程，将电力、通信等7类管线纳入地下管廊。新建人防工程7个，提高城市综合防护能力。规范共享单车、电动车管理，努力提升城市管理水平。完善住宅专项维修资金管理办法和物业企业考评办法，推进物业企业专业化发展，提高物业管理服务质量。大力发展装配式建筑，绿色建筑占新建建筑比例达到40%。

推动城乡融合发展。全面落实“产业兴旺、生态宜居、乡风文明、治理有效、生活富裕”乡村振兴战略总要求，依托美丽乡村、特色小镇、危房改造，推动水、电、路、气、暖等基础设施和优质公共服务向农村覆盖延伸。加快实施红寺堡供水改造二期、惠安堡中心卫生院提标改造等项目，尽快缩小山川基本公共服务差距。新建改建农村公路300公里，罗山大道石家窑至石沟驿段建成通车。推进城乡客运一体化，合理配置现有公交运力，优化公交资源线路。稳步推进大水坑、金银滩、韦州等5个特色小镇培育，改造危窑危房3000户以上，建设美丽村庄20个，农村改厕3000户。深入开展农村人居环境综合治理行动，争取市场化运营全覆盖。加快推进城乡水务一体化，确保农村饮水安全。加快树立文明乡风，制定实施《吴忠市移风易俗奖惩办法》，评选表彰一批优秀红白理事会、移风易俗示范户、星级文明户。

四、更加注重脱贫富民，切实保障改善民生

坚持以人民为中心的发展思想，进一步织牢民生保障网，切实解决人民群众的烦心事、难心事、闹心事，让人民群众有更多的获得感、安全感和幸福感，在脱贫富民上展现新作为。

打好精准脱贫攻坚战。聚焦盐同红交界处集中连片贫困地区，打好四场硬仗、实施八项工程、落实十大行动，脱贫不脱政策、脱贫不脱项目、脱贫不脱帮扶、脱贫不脱监管，完成22个贫困村、14个深度贫困村稳定退出和2.3万人脱贫以及206个脱贫出列村的巩固提升任务。出台支持红寺堡区、同心县脱贫摘帽政策措施，举全市之力帮助红寺堡区、同心县打赢脱贫攻坚战。深化产业扶贫，全面落实扶持优势特色农业产业发展政策措施，走规模经营与精准到村到户相结合路子。大力发展电商扶贫、乡村旅游扶贫，稳步推进扶贫车间建设，不断拓宽群众增收渠道。加大金融扶贫力度，实现有劳动能力、有贷款意愿建档立卡贫困户扶贫小额信贷“应贷尽贷”。重视提高贫困人口知识素质，大力强化劳动技能培训，年内培训建档立卡劳动力1万人以上。围绕基础设施和公共服务，完善盐同红交界处集中连片贫困地区“七纵六横”交通路网，实施刘家沟水库扩容蓄水、盐池县中南部水源工程，贫困村光纤宽带、4Ｇ网络实现全覆盖。坚持脱贫速度服从脱贫质量，既不降低标准、影响质量，也不随意调高标准、脱离实际，确保贫困县摘帽符合“三率一度”标准，贫困户退出符合“两不愁、三保障”要求。加大对收入水平略高于建档立卡贫困户群体的支持力度，防止返贫和产生新的贫困。持续深化脱贫攻坚作风建设，全面压实脱贫攻坚主体责任、帮扶责任、督查责任，坚决杜绝急功近利、弄虚作假，确保扶贫工作务实，脱贫过程扎实，脱贫结果真实。

加快发展社会事业。落实教育优先发展战略，增加学前教育、农村贫困地区儿童早期发展、职业教育等投入。开展教师素质和教育质量“双创优”行动，完善教师补充、交流、培训机制，提高教育教学水平。加快义务教育薄弱学校标准化建设，新建学校（含幼儿园）17所，改扩建6所，“大班额”比例控制在5%以内。深入推进“互联网+教育”，建设“在线课堂”教室150间，组建银川都市圈利青发展合作体19个，跨区域合作办学学校扩大到100所。坚持产教融合，扩大校企合作，提高职业教育专业建设水平。深化“互联网+医疗健康”，完善分级诊疗服务体系，大力开展双向转诊、远程医疗等服务。积极推进县级医院标准化建设，加快市人民医院创建三级甲等医院步伐，深化与北京友谊医院、厦门大学附属第一医院等知名院校、医疗团队的合作。认真践行社会主义核心价值观，以庆祝中华人民共和国成立70周年为主题，推出一批讴歌新时代、弘扬主旋律、凝聚精气神的精品力作。实施文化惠民工程，力促青铜峡市文化馆、图书馆，盐池县图书馆投入使用，提升村级综合文化服务中心功能。组织送戏下乡演出300场次、“滨河百姓大舞台”广场演出50场次。高标准筹办自治区第十五届运动会、第八届宁夏黄河金岸（吴忠）国际马拉松赛、2019年全国沙滩排球巡回赛（宁夏吴忠站）等体育赛事。加快创建全国文明城市，努力建设志愿之城，全面提升城市品位。加强全民科学素质工作，大力提升公民科学素养。依法加强统计监管，高质量开展第四次全国经济普查。加强国防教育、国防动员和国防后备力量建设，推动军民融合发展，巩固军政军民团结的良好局面。

提升社会保障水平。深入落实稳就业各项政策措施，扩大就业数量，提升就业质量，年内新增就业1.2万人，农村劳动力转移就业18万人。落实更大规模的减税降费政策，充分发挥创业孵化园、创业基地示范带动引领作用，鼓励支持高校毕业生、复转军人、返乡农民工自主创业，建设市区人力资源交易中心。实施全民参保登记，“两险”参保率稳定在90%、98%以上。建成医保智能监控系统，推进社保登记“网上申报”改革，年内80%参保单位完成网上申报。大力发展残疾人事业，建成运营残疾人康复中心，提高残疾康复服务水平。健全社会救助济困体系，深化低保专项治理。加强敬老院、老年活动中心、老年饭桌等养老服务管理，建立以居家为基础、社区为依托、养老机构为补充的养老服务体系，推进养老服务质量稳步提升。扎实做好退役军人服务管理工作，维护保障退役军人合法权益。

五、更加注重生态立市，持续改善生态环境

深刻领会习近平生态文明思想，牢固树立“绿水青山就是金山银山”的理念，坚决打好污染防治攻坚战，不折不扣抓好中央环保督察反馈问题和“绿盾”专项行动排查问题整改，促进经济社会发展和生态文明建设相辅相成、相得益彰，在绿色发展上培育新优势。

加强生态建设保护。全力创建国家森林城市，实施大规模国土绿化行动，持续抓好黄河生态修复、退耕还林、防沙治沙等工作，完成营造林25.85万亩，全市森林覆盖率提高到17%。实施市区道路绿网、利青生态廊道2个城市绿化重点建设工程，新建改造城市绿地1300亩。加强自然保护区建设和管理，深入排查整治罗山、哈巴湖、青铜峡库区突出环境问题，坚决惩处破坏自然保护区生态环境的违法违规行为。加强野生动物保护，深化禁牧封育成果，构建有利于生物多样性的生态环境。

打赢蓝天保卫战。推行空气质量精细化管理，建设空气质量监测网格及生态环境数据平台，空气质量优良天数比例稳定在80%以上。加快城市周边村、城中村“煤改气”“煤改电”进程，完善清洁煤配送体系，继续推进燃煤锅炉除尘脱硫脱硝改造，提升污染防治水平。开展工业企业全面达标排放行动，实施金昱元自备电厂超低排放等项目。持续开展工业炉窑整治和挥发性有机物治理，彻底整治散乱污企业，对达不到要求的依法停产关闭。强化扬尘管控巡查，加强建筑工地、非煤矿山、城市裸露地扬尘整治。严格执行“国五标准”，有效治理柴油货车污染。推广秸秆制作生物质燃料，扩大秸秆还田、高温堆肥覆盖面，全年秸秆资源化利用率达到90%以上。

打好碧水保卫战。全面落实“水十条”，集中力量打好新时代黄河保卫战。深入推进河（湖）长制，加大河湖水环境综合治理力度，完成河湖水域岸线划界确权工作。巩固入黄排水沟治理成果，发挥好“两沟”入黄口人工湿地作用，确保入黄排水沟水质达到IV类、黄河吴忠段断面水质稳定在II类优水质。加快实施第一污水处理厂尾水人工湿地及金积工业园区、太阳山开发区污水处理厂人工湿地建设项目，建成第四、第五污水处理厂。启动同心张家山等12个小流域治理项目，完成罗家河水质改善、青铜峡东部水系连通工程。实施五里坡四期水资源配置、盐池德胜墩水资源综合利用等工程，争取盐同红革命老区供水工程开工建设。

推进净土保卫战。全面落实“土十条”，开展生态红线勘界定标和土壤污染状况详查工作，严格建设用地准入管理，防止新的污染。加强涉重金属行业污染防治，全程监管重点工业行业危险废弃物处置。加大农用地保护力度，扎实开展化肥农药减量、畜禽粪污治理、农膜回收处理等农业面源污染治理。

六、更加注重改革创新，不断激活发展动力

全面深化改革创新，健全与经济高质量发展相适应的体制机制，更加注重优化发展环境，不断激发全社会的创造动力和发展活力。

深化重点领域改革。深化农村改革，全面完成农村“房地一体”、土地承包经营权确权登记颁证，落实好第二轮土地承包到期后再延长30年政策。深入推进同心县农村土地承包经营权抵押贷款试点和利通区农村集体产权制度改革试点，加快县级农村产权交易服务中心建设，扶持110个“空壳村”实施村集体经济项目。鼓励支持利通区探索整治农村“空心房”。实施水流产权、农业水价综合改革，推进利通区、红寺堡区现代化生态灌区试点。加强财政资金预算绩效管理，推进非税收入征管体制改革。构建国有资产报告机制，建立国有资产数据库和信息共享平台。深化国企改革，重点在资本运营、混合所有制等方面取得新突破。组建运营大数据公司、招商引资服务机构、社会信用体系建设服务中心。深化“三医联动”改革，推进县域综合医改试点。落实公立医院人事薪酬制度改革，探索推进编制、岗位“县管乡用”。进一步落实按病种支付方式改革，控制医疗费用不合理增长，公立医院药占比控制在30%以下。组织外贸进出口企业参加德国有机食品展等国际展会20场次，全年进出口总额增长10%以上。进一步扩大35个展示展销中心销售推介成果，支持发展网络营销、社区营销、互联网+餐饮等模式，年内发展二级销售网点50个，确保全年销售额达到4亿元。深化与宁波等友好城市的交流合作，推动形成宽领域、多层次、全方位合作格局。

增强科技创新能力。大力实施创新驱动战略，全面推进沿黄科技创新改革试验区建设，力争全市R&D投入在上年基础上增长30%以上、投入强度达到1.3%以上。积极推进沿黄生态经济带建设行动计划，谋划实施一批科技含量高、经济效益好、资源消耗低、环境污染少的清洁生产、污染治理、低碳循环项目，引导支持企业加快研发生态产品和绿色产品。围绕高端智能控制阀、精密汽车零部件、现代纺织等产业，建设公共技术服务平台。积极打造科技企业孵化器，加快建设中国智能装备制造（仪器仪表）产业知识产权运营中心和吴忠技术市场。深化产学研合作，加快建设中国（宁夏）奶业研究院，支持吴忠仪表与清华大学、多伦多大学合作建设流体控制装备及技术国家重点实验室。新培育国家高新技术企业4家以上、自治区农业高新技术企业3家以上、自治区科技小巨人企业3家以上、自治区科技型中小企业20家以上。加快推进金积工业园区创建自治区级高新技术产业开发区、吴忠国家农业科技园区创建自治区级农业高新技术产业园区。加快“两化”深度融合，新增全国两化融合贯标试点企业1家、自治区智能工厂（数字化车间）2家。促进金融与科技深度融合，继续推进科技型中小微企业风险补偿专项资金试点工作，扩大科技担保基金覆盖面。

加强人才队伍建设。认真落实《引进和培养人才激励办法》《加快产业人才高地建设助推创新驱动发展战略的实施办法》，加快建设健康养生、装备制造、现代纺织、全域旅游等7个产业人才高地。深入实施企业家素质提升行动计划和人才培养工程，培训、培养高层次人才50名、企业经营管理人才500名、农村实用人才1500名、专业技术人才3500名。加大人才引进力度，创新人才评价、激励和服务机制，建立人才公寓，让各类人才引得进、留得住、干得好。鼓励企事业单位以挂职兼职、科技攻关等方式柔性引才，支持国内、海外科技创新团队在我市创办科技型企业。加强人才创新平台建设，新建企业博士工作站2个、院士工作站和专家服务基地3个。

大力优化营商环境。深化“放管服”改革，持续推进“互联网+政务服务”，加快建设“一库、一网、一端”，网上可办率不低于70%，“一窗”分类受理达到70%以上。大力优化审批程序，扎实推进“证照分离”，投资项目全流程审批压缩至110天，网上办照不超过1个工作日。设立“一站式”综合服务窗口，实现“前台综合受理、后台分类审批、统一窗口出件”。全面落实吴忠市优化营商环境25条措施，努力打造“标准高、水平好、审批少、收费低、速度快、服务优”的营商环境。依托城市大数据运营中心，加快政府审批便企便民系统的互联互通，让信息多跑路，让群众少跑腿。大力支持民营经济发展，认真贯彻落实习近平总书记重要讲话精神和自治区《关于促进民营经济健康发展的若干意见》以及“降成本30条”等惠企政策，帮助企业降本增效。加快搭建政商沟通平台，构建“亲”“清”新型政商关系。依法保护民营企业家的人身安全和财产安全，鼓励引导规范经营，营造公平竞争市场环境。

七、更加注重创新治理，维护社会大局和谐稳定

着力推进社会治理体系和治理能力现代化，维护社会和谐稳定，巩固安定团结社会局面，为全市经济社会发展营造良好社会环境。

不断完善社会治理体系。全面落实普法责任制，大力推进“法律八进”，加强法制教育，让尊法学法守法用法成为全社会的共同追求和自觉行动。完善公共法律服务体系，建设智慧法律服务平台，实行“一站式、综合化”便民服务。探索建立“互联网+法律”服务模式，逐步形成网上网下相融合的公共法律服务格局。积极推广“枫桥经验”，深化人民调解“四张网”建设，提升人民调解工作水平。加强基层社会治理，不断提升城乡社区治理水平。建立“信用吴忠”平台，积极创建社会信用体系示范城市。

切实加强民族宗教工作。全面贯彻党的民族政策，开展“统战工作规范提升年”活动，推进马克思主义民族观宗教观和党的民族宗教法规政策宣传教育。深化民族团结创建行动，推进创建工作走实、走深、走细，在民族团结上创造新经验。坚持宗教中国化方向，深入开展“四进”宗教场所等活动，完善宗教事务管理量化体系，推行宗教事务规范化精细化管理，落实乡村两级宗教工作责任制。深入开展宗教领域突出问题整治，全面完成违规建设宗教场所问题整改，依法严厉打击非法宗教活动，确保宗教领域和顺平静。

全力维护社会和谐稳定。推动平安建设重心下移，巩固提升全国社会治安综合治理优秀市成果。依法惩治各类违法犯罪行为，深入推进扫黑除恶专项斗争，深挖彻查黑恶势力“关系网”“保护伞”，保障人民群众生命财产安全。依法规范信访行为和信访秩序，积极主动化解信访积案。积极创建全区安全发展示范城市，强化应急管理，推进安全监管信息化建设，有效提升应对和处置各类突发事件能力水平。落实党政同责“四个最严”要求，不断加强食品药品安全监管，强化风险全程管控，保障人民群众“舌尖上的安全”。

八、更加注重依法行政，全面提升政府工作水平

2019年是市县党政机构改革完成年和新机构履职尽责的第一年。面对新职能、新使命、新任务，我们要坚持党的领导，牢固树立“四个意识”，坚定“四个自信”，坚决维护习近平总书记党中央的核心、全党的核心地位，坚决维护党中央权威和集中统一领导，切实加强政府自身建设，实字当头、干字为先，以勇于担当、善于担当的“宽肩膀”“铁臂膀”落实各项工作，提升履职水平，为人民群众提供更加优质高效的服务。

始终坚持依法行政。加强法治政府建设，以法治思维、法治方式推进工作。完善政府部门权责清单，全面落实市场准入负面清单，凡是市场能自主调节的就由市场调节，凡是企业能干的就交给企业干。深入开展行政执法全过程记录、行政执法公示、重大执法决定法制审核“三项”制度，提高执法能力和水平。坚持科学民主决策，自觉接受人大法律监督、政协民主监督和社会公众监督。认真办理人大建议和政协提案，坚持重大事项向人大报告、向政协通报制度，主动配合人大开展工作评议、政协实施民主协商。加大政府信息公开力度，强化舆情分析应对，主动回应社会关切，接受人民监督。认真贯彻实施新《公务员法》，全力打造忠诚干净担当的公务员队伍。

始终坚持高效尽责。调整优化政府机构设置和职能配置，完成市县机构改革，积极构建系统完备、科学规范、运行高效的机构职能体系，做到政令畅通、执行有力。主动加强学习，克服“本领恐慌”，努力增强“八种本领”。坚持以目标倒逼进度、以督查强化落实、以绩效论英雄，确保各项工作高效运行。坚决克服官僚主义、形式主义，整治“痕迹”管理问题，坚决杜绝重“痕”不重“绩”，留“痕”不留“心”，切实为基层减负。严格执行“三个区分开来”，健全完善正向激励、容错纠错机制，严厉整肃庸政懒政怠政行为，严肃治理政务失信。

始终坚持勇于担当。大力发扬振奋精神、实干兴宁的作风，切实增强创新意识、实干意识和服务意识，以“踏石留印、抓铁有痕”的劲头全面提高工作质量。强化担当意识，敢抓敢管、动真碰硬，遇到矛盾不回避、面对困难不退缩，不怕接硬任务、主动啃硬骨头，在攻坚克难中诠释忠诚、在推动发展中体现担当。对中央、自治区和市委的决策部署，坚决执行、迅速落实。

始终坚持勤政廉洁。认真开展“不忘初心、牢记使命”主题教育，坚定理想信念，更加自觉地践行党的宗旨。贯彻全面从严治党要求，认真学习领会习近平总书记在十九届中央纪委三次全会上的重要讲话精神，贯彻落实“五个必须”和“五个确保”要求，严格落实党风廉政建设“一岗双责”和领导干部廉洁从政各项规定，始终把纪律和规矩挺在前面。坚决贯彻落实中央八项规定精神和区、市相关规定，一以贯之整治“四风”。加强政风行风监督，提升窗口单位服务质量。全力抓好中央、自治区巡视和市委巡察、督查问题整改，以整改成效促工作提升。强化审计监督和成果运用，规范政府权力运行，严控“三公”支出。始终保持勤政廉政，严格执行廉洁自律准则和纪律处分条例，巩固发展反腐败斗争压倒性胜利，确保干部清正、政府清廉、政治清明。

各位代表，实干成就梦想，奋斗再铸辉煌。让我们紧密团结在以习近平同志为核心的党中央周围，在自治区党委、政府和市委的坚强领导下，勇于担当，锐意进取，开拓创新，真抓实干，努力实现经济繁荣、民族团结、环境优美、人民富裕，确保与全国全区同步建成全面小康社会，为“建设美丽新宁夏、共圆伟大中国梦”而努力奋斗，以优异成绩庆祝中华人民共和国成立70周年！
